# Development and validation of a novel overhead method for anteroposterior radiographs of fractured rat femurs

**DOI:** 10.1038/s41598-024-56238-4

**Published:** 2024-03-06

**Authors:** Yosuke Sato, Takashi Tagami, Toshio Akimoto, Toru Takiguchi, Yusuke Endo, Takeshi Tsukamoto, Yoshiaki Hara, Shoji Yokobori

**Affiliations:** 1 Department of Emergency and Critical Care Medicine, Nippon Medical School, 1-1-5 Sendagi, Bunkyo-ku, Tokyo, Japan; 2 Department of Emergency and Critical Care Medicine, Nippon Medical School, Musashikosugi Hospital, 1-396 Kosugimachi, Nakahara-ku, Kawasaki, Kanagawa 211-8533 Japan; 3 Division of Laboratory Animal Science, Nippon Medical School, Tokyo, Japan; 4 Department of Emergency and Critical Care Medicine, Nippon Medical School, Chibahokusoh Hospital, Inzai, Japan

**Keywords:** Experimental models of disease, Fracture repair

## Abstract

We aimed to establish a new method of obtaining femur anteroposterior radiographs from live rats. We used five adult male Sprague–Dawley rats and created a femoral fracture model with an 8 mm segmental fragment. After the surgery, we obtained two femoral anteroposterior radiographs, a novel overhead method, and a traditional craniocaudal view. We obtained the overhead method three times, craniocaudal view once, and anteroposterior radiograph of the isolated femoral bone after euthanasia. We compared the overhead method and craniocaudal view with an isolated femoral anteroposterior view. We used a two-sample t-test and intraclass correlation coefficient (ICC) to estimate the intra-observer reliability. The overhead method had significantly smaller differences than the craniocaudal view for nail length (1.53 ± 1.26 vs. 11.4 ± 3.45, *p* < 0.001, ICC 0.96) and neck shaft angle (5.82 ± 3.8 vs. 37.8 ± 5.7, *p* < 0.001, ICC 0.96). No significant differences existed for intertrochanteric length/femoral head diameter (0.23 ± 0.13 vs. 0.23 ± 0.13, *p* = 0.96, ICC 0.98) or lateral condyle/medial condyle width (0.15 ± 0.16 vs. 0.13 ± 0.08, *p* = 0.82, ICC 0.99). A fragment displacement was within 0.11 mm (2.4%). The overhead method was closer to the isolated femoral anteroposterior view and had higher reliability.

## Introduction

Plain radiography is the classic technique and remains the standard tool for evaluating bone union^1^. The last few decades have witnessed significant improvements in other imaging techniques, such as ultrasonography and micro-computed tomography, to evaluate fracture morphology and the healing process. Although each technique has its strengths, several weaknesses and limitations have been identified. Ultrasonography is a non-invasive evaluation method for bones and soft tissues, such as the muscles, tendons, ligaments, nerves, and blood vessels. However, the accuracy and precision of the imaging are largely dependent on the operator^2,3^. Micro-computed tomography (CT) poses a major challenge in evaluating bone morphology owing to hard metal artifacts, particularly in fracture models with implanted bones^4^. Therefore, plain radiographic evaluations are rapid and reproducible and are still considered the best and first step in assessing the bone healing process^5^. However, it is important to note that these evaluations also have some limitations due to the projection of the sample. For example, the orientation of the femur during scanning might alter the observed angle of inclination.

Two-directional plain radiographic imaging is a standard practice for assessing bone union in rats^5–7^. While the method for capturing lateral radiographic images of a living rat's femur is well-established, a technique for obtaining anteroposterior radiographs of a living rat's femur has not yet been developed. Historically, two-directional imaging evaluations were only performed on deceased rat femur bones or by pathological methods after sacrificing the rats^8–10^. If images can be obtained in the anteroposterior direction without sacrificing the animal, it would allow for the continued use of the same rats over an extended period. This advancement would enable the application of the classic, well-established two-directional method for evaluating bone union in living rats^5–7^. Furthermore, the number of rats required for the experiment can be reduced. Additionally, it is desirable to use a method that does not affect the intermediate bone fragments during imaging because some experiments use femoral comminuted fracture models or models with segmental bone fragments^11^.

The craniocaudal view^12^ is a well-established technique for obtaining femoral anteroposterior views in dogs. However, this technique is not commonly used in living rats because it is difficult to achieve full extension of both hip and knee joints, as is typically done in dogs. This study aimed to develop and validate a novel technique for obtaining anteroposterior radiographs of fractured femurs in living rats with a free fragment. This technique is specifically designed to overcome the inherent limitations of traditional radiographs.

## Methods

This study was approved by the Animal Experiments Ethical Review Committee of Nippon Medical School (No. 2021-061). All methods were performed in accordance with the relevant guidelines and regulations. We report the current study in accordance with ARRIVE guidelines.

### Animal

Ten-week-old male Sprague–Dawley rats (n = 5, Shizuoka Laboratory Animal Center, Japan) weighing 356–419 g were used in this study. Animals were fed a commercial diet (MF; Oriental Yeast Co., Tokyo, Japan) and tap water ad libitum. They were housed in an air-conditioned room (21 ± 2 °C, 50–60% relative humidity, and lights on for 14 h per day from 6:00 to 20:00).

### Surgical anesthesia and euthanasia protocol

Each rat was anesthetized using 2–3% isoflurane (VTRS, VIATRIS, USA) in oxygen. For preemptive analgesia, buprenorphine (0.01 mg/kg, 0.3 mg/1.5 ml, NISSIN, Japan) was subcutaneously administered. The rats were euthanized with an overdose of isoflurane in the laboratory following the acquisition of necessary radiographs. We adjusted the isoflurane flow concentration to 5%, and exposure to isoflurane continued until 2 min after the cessation of breathing.

### Radiography (Fig. 1, 2, 3)

**Figure 1:**
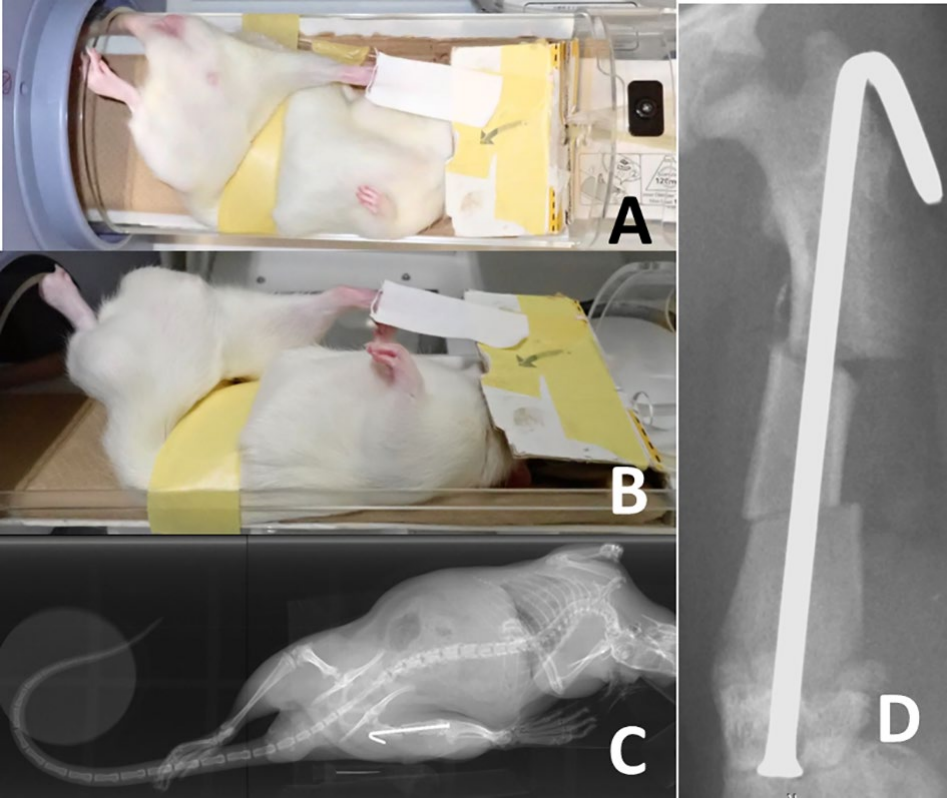
Overhead method. In the supine position, the rat was positioned on a cardboard sheet, and a pulley system was created by placing a strip of tape over the lower abdomen. The rat’s affected limb was taped to the soles and pulled straight towards the head. The key was to pull firmly to ensure the buttock floated approximately 2 cm from the dorsal margin of the base of the tail to the ground. Subsequently, we fine-tuned the rotation to align the patella frontally, and radiography was performed. (**A**) Frontal view: A pulley was established on the cranial side of the hip center. (**B**) Lateral view. (**C**) The entire radiograph obtained in this position. (D) Enlarged view of the femur extracted from the complete radiograph (**C**).

**Figure 2:**
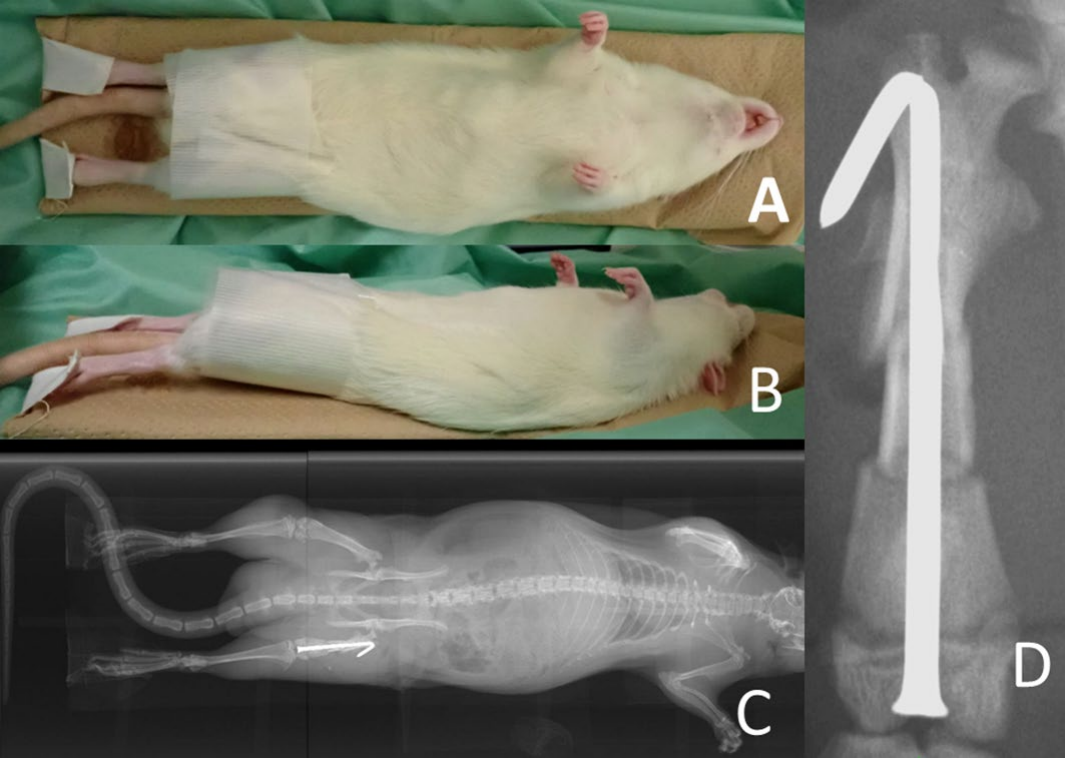
Craniocaudal view. In the supine position, a rat was positioned on a cardboard sheet. To ensure a frontal alignment of the patella, both knees were securely fastened with tape, pulled caudally with manual maximum force, and fixed with tape. (**A**) Frontal view: Both knees were rotated internally. (**B**) Lateral view: No additional support or restraints were applied to hold the trunks. (**C**) The entire radiograph obtained in this position. (**D**) Enlarged view of the femur obtained from the complete radiograph (C).

**Figure 3:**
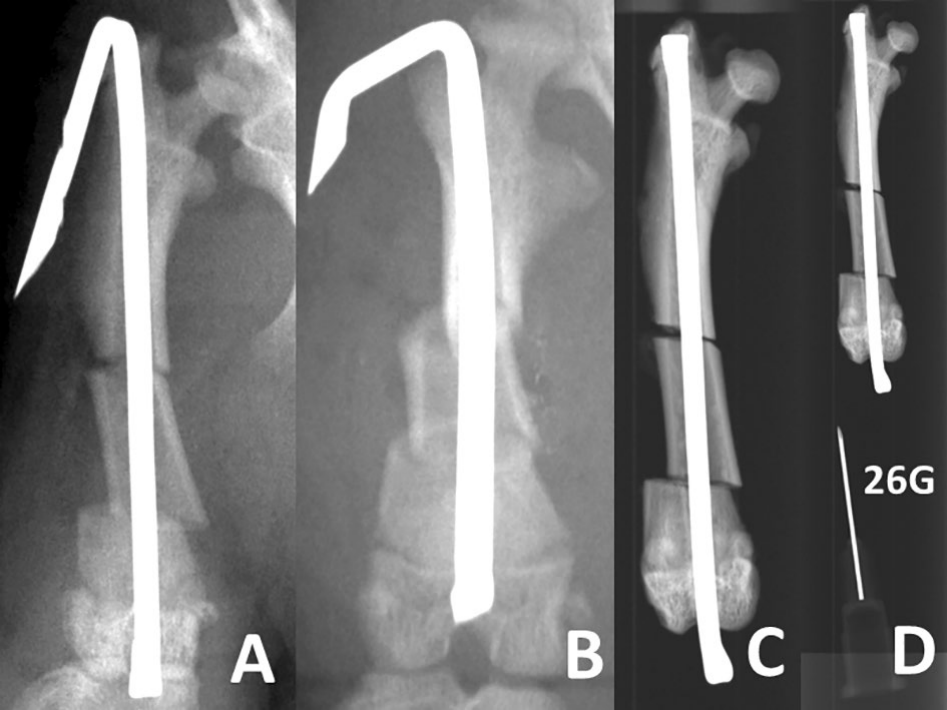
Representative images of three anteroposterior views. These images depict representative views of the same rat. (**A**) Overhead method. (**B**) Craniocaudal view. (**C**) Isolated femoral anteroposterior view. (**D**) The 26G needle along with the femur was radiographed to correct the magnification concerns associated with the overhead method, craniocaudal view, and isolated femoral anteroposterior view.

Micro-computed tomography (Latheta LCT-200, Hitachi Aloka Medical, Mitaka, Tokyo, Japan) was used to obtain the images. All image analyses were performed using the OsiriX MD software (vers.13.0.1, Pixmeo, Switzerland).

We obtained two anteroposterior images as follows.

1. Overhead method (Fig. 1): In the supine position, we placed the rat on a cardboard sheet and created a pulley by placing a piece of tape over the rat's lower abdomen. The rat was taped onto the sole of the affected limb and pulled straight toward the head. The other limb was not fixed. We then fine-tuned the rotation as the patella faced directly forward and radiography was performed.
2. Craniocaudal view (Fig. 2): In the supine position, both knees were tightened, and both femurs were rotated inward so that the patellae lay over the patellar grooves^12^. Subsequently, we manually pulled both lower limbs straight caudally with maximum force and taped them.
3. Isolated femoral anteroposterior view (Fig. 3): Following euthanasia, the right hind femur, along with all surrounding soft tissue was removed. For radiographic examination, the femur was positioned so that the inferior surfaces of both femoral condyles were parallel to the ground. This view was then used as the gold standard for assessing the anteroposterior view.

### Operative methods

**Figure 4:**
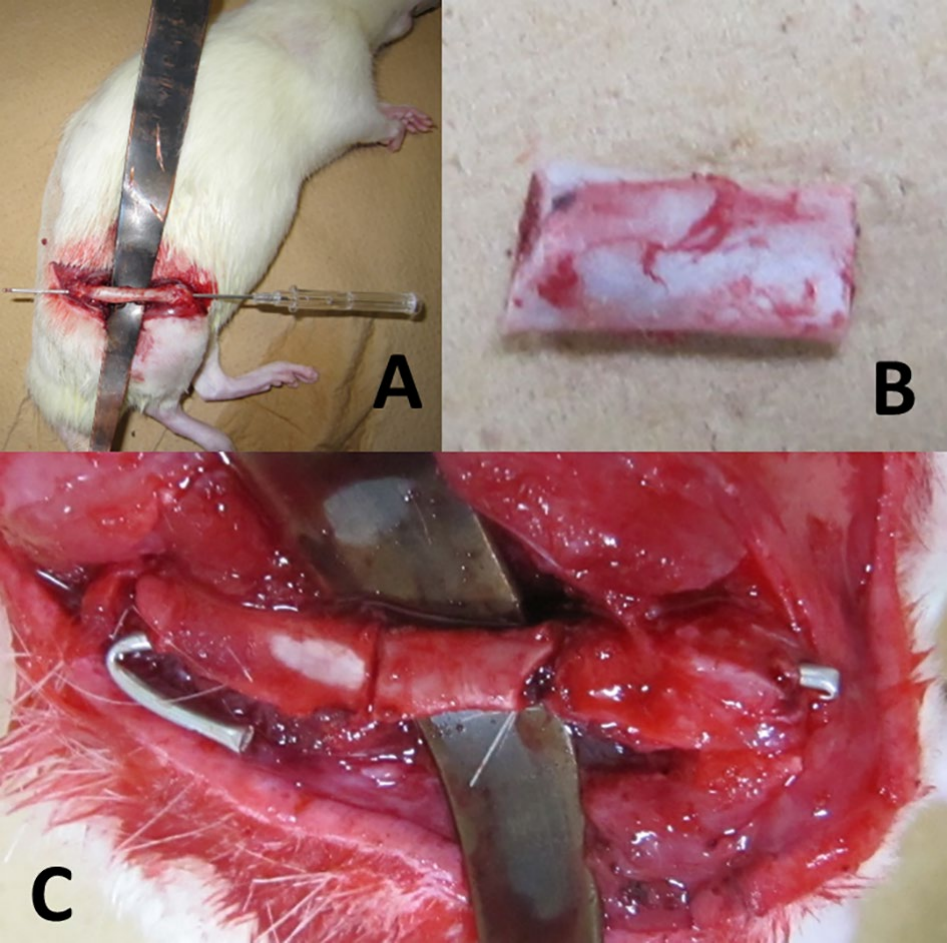
Femur fracture model with a segmental free fragment. After the diaphysis was exposed, a cut was made at a distance of 8 mm from the third trochanter. We created a segmental free fragment and fixed it using a 16G intravenous cannula inner needle. The base of the needle was cut sharply, bent briefly and dorsally, and inserted deeply into the bone to prevent dislodgment. The tip of the needle on the other side was bent along the diaphysis. (**A**) Intraoperative view of the exposed femoral diaphysis of a rat with retrograde insertion of a 16G inner needle. Subsequently, the needle was removed temporarily before creating the segmental fragment. (**B**) An 8 mm segmental free fragment was formed by completely removing the soft tissues. (**C**) The free fragment was returned to its original position and fixed with a needle.

We created a femoral fracture model with a large segmental free fragment (Fig. 4).

A skin incision was made in the right femur. The subcutaneous fascia lata was cut, and the quadriceps was bluntly dissected. We measured the femoral bone length from the greater trochanteric tip to the cartilaginous surface of the femur. The bone was cut just below the third trochanter and 8 mm below the third trochanter using a diamond disk (Dremel Lite™, Bosch Power Tools B.V., Tokyo, Japan). A bone fragment 8 mm in length was created, and the soft tissue was completely peeled off of this fragment. The muscles attached to the third trochanter were preserved. Subsequently, an inner needle of a 16G intravenous cannula (Surflo™, SR-OT1664C, TERUMO, Tokyo, Japan) was inserted from the intercondylar sulcus to the free fragment and the proximal diaphysis. An inner needle was used as a nail. This model did not have rotational stability but had angular and longitudinal stability. We obtained anteroposterior radiographs of live rats under anesthesia using the two methods. First, the overhead method was performed three times to measure reliability. Subsequently, a craniocaudal view was taken. The rats were then euthanized. The hind right femoral bone was removed, and the isolated femoral anteroposterior view was then obtained. We compared the overhead method and craniocaudal view with the isolated femoral anteroposterior view.

### Outcome measure (Fig. 5)

**Figure 5:**
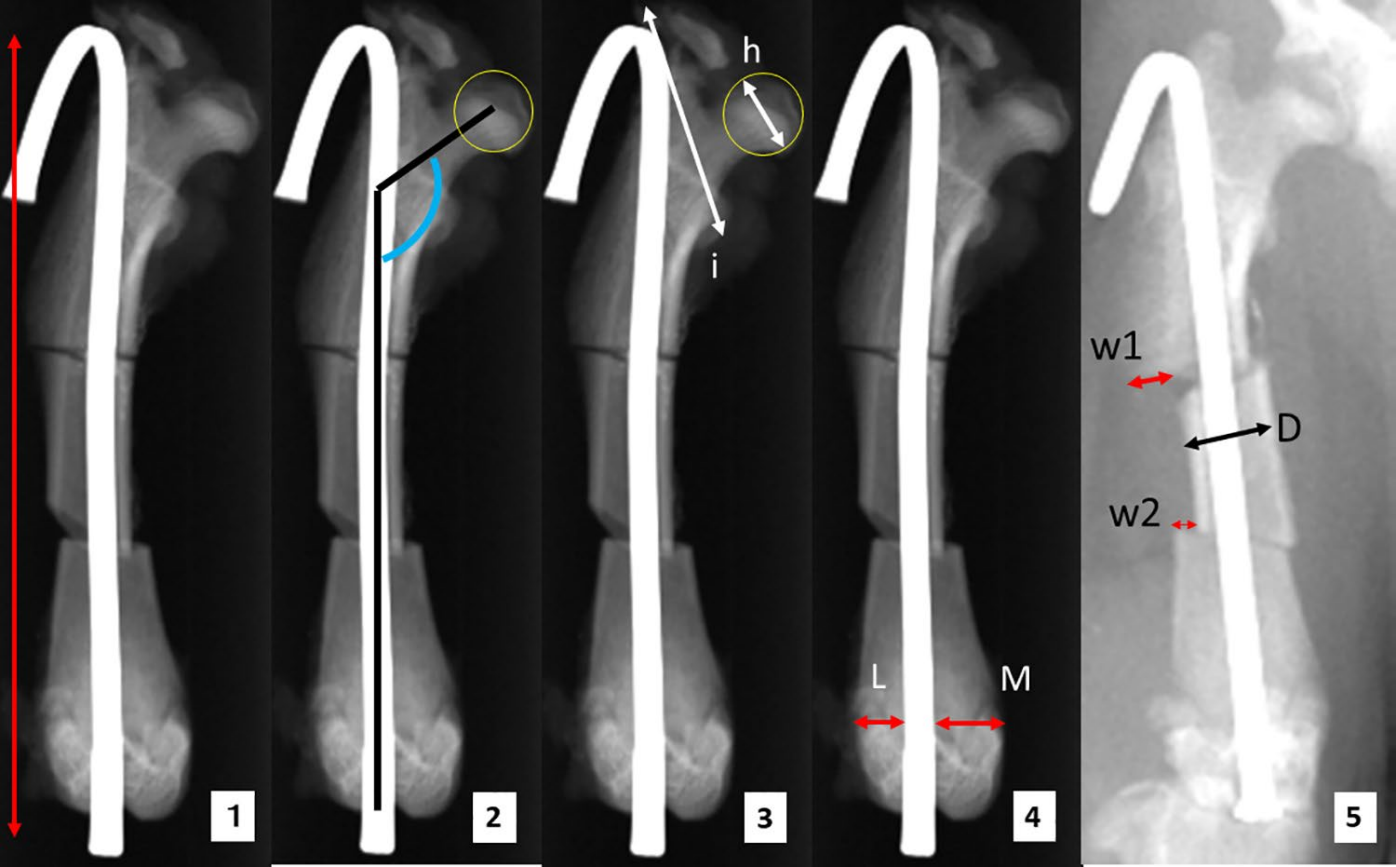
Measurement items. This figure outlines the specific measurements recorded during the study: (**1**) Nail length: The nail length between the folded points. (**2**) Neck shaft angle: The angle between the longitudinal femoral shaft axis and the femoral head-neck axis. The femoral head-neck axis was defined by a line bisecting the femoral neck through the center of the femoral head. The center of the femoral head was defined as the center of a maximum circle drawn around the femoral head. The longitudinal femoral shaft axis was determined as the long axis of the intramedullary nail. (**3**) Intertrochanteric length (i)/femoral head diameter (h): Intertrochanteric length (i) was defined as the longest length between the tip of the lesser trochanter and the tip of the greater trochanter. Femoral head diameter (h) was defined as the diameter of a maximum circle drawn around the femoral head. (**4**) Lateral condyle width (L)/internal condyle width (M): At the level of the epiphysis line, we measured the lateral condyle width (L) as the length between the outermost edge of the lateral cortex to the lateral nail line and the internal condyle width (M) as the length between the outermost edge of the medial cortex to the medial nail line. We then divided the value of L by the value of M. (**5**) Displacement of the free fragment: We defined the proximal cortical steps between the proximal diaphysis and the free fragment (W1) and the distal cortical step between the distal diaphysis and the free fragment (W2). W1 and W2 at the first and third times of the overhead method were measured. We divided the absolute value of W1 + W2 at the first time measurement − W1 + W2 at the third time measurement by the width at the center of the fragment (D).

To examine how closely the overhead method and the craniocaudal view align with the gold standard, the isolated femoral anteroposterior view, we measured the nail length, neck shaft angle, intertrochanteric length/femoral head diameter, lateral condyle width/medial condyle width, and free fragment displacement between the first and third measurements/width of the center of the free fragment. The definitions are as follows:

1. *Nail length* This was defined as the distance between the most proximal bending point and the most distal bending point of the needle as an intramedullary nail.
2. *Neck shaft angle* This was defined as the angle between the longitudinal femoral shaft axis and the femoral head-neck axis, as in the human femoral neck-shaft angle. The femoral head-neck axis was defined by a line bisecting the femoral neck through the center of the femoral head. The center of the femoral head was defined as the center of a maximum circle drawn around the femoral head. The longitudinal femoral shaft axis was determined as the long axis of the intramedullary nail.
3. *Intertrochanteric length/femoral head diameter* Intertrochanteric length was defined as the longest length between the tip of the lesser trochanter and the tip of the greater trochanter. The femoral head diameter was defined as the diameter of a maximum circle drawn around the femoral head. We then calculated the intertrochanteric length divided by the femoral head diameter.
4. *The lateral condyle width/the internal condyle width* At the epiphysis line, we measured the lateral condyle width (i.e., the length between the outermost edge of the lateral cortex to the lateral nail line) and the internal condyle width (i.e., the length between the outermost edge of the medial cortex to the medial nail line). We then calculated the lateral condyle width divided by the internal condyle width.
5. *Displacement of the free fragment* We defined the proximal cortical step (i.e., the absolute distance between the outermost margin of the cortex on the proximal diaphyseal side and the outermost margin of the cortex of the free fragment at the proximal osteotomy site) and the distal cortical step (i.e., the absolute distance between the outermost margin of the cortex on the distal diaphyseal side and the outermost margin of the cortex of the free fragment at the distal osteotomy site). We calculated the sum of the proximal cortical step and the distal cortical step at the first and third times of the overhead method. The difference between the sum of the first time and the sum of the third time was defined as the displacement. The displacement was divided by the width at the center of the fragment to adjust for the magnification.

Nail length was measured as an index of hip angulation and femur magnification; neck shaft angle and intertrochanteric length/femoral head diameter were the indices of proximal rotation, and the lateral condyle width/medial condyle width was the index of distal rotation. The displacement of the free fragment was measured to evaluate the stress shifting of the free fragment when we repeated the overhead method. Each slightly different magnification was corrected by taking radiographs along with a 26G needle (NN-2613S, TERUMO, Tokyo, Japan) (Fig. 3).

### Statistics and data analysis

To validate our new measurement technique, it's essential to evaluate both the accuracy and precision of the measurements. Accuracy is defined as how closely a measurement aligns with the true or actual value, while precision refers to the consistency of repeated measurements with one another^13,14^.Except for the displacement of the free fragment, the difference between the overhead method (first time) and the isolated femoral anteroposterior view, craniocaudal view, and isolated femoral anteroposterior view was calculated for each item. We used a two-sample t-test to compare the differences for the evaluation of accuracy. Precision refers to the consistency of repeated measurements with each other. We assessed the precision of the overhead method by using the Intraclass Correlation Coefficient (ICC), following confirmation of its high accuracy. We used the ICC (1,1) to assess the intra-observer reliability of the overhead method. The detailed mathematical principles and concepts of ICC are discussed elsewhere^14–20^. Briefly, the ICC assesses the agreement of quantitative measurements in consistency and conformity^19^ and is the proportion of the total variance attributable to true differences between variables. To assess intra-observer reliability, ICC (1,1) was used for reliability over an average of three measurements. In general, the ICC (ρ) ranges from 0 to 1 and characterizes values of reliability as follows: slight (0.0–0.20), fair (0.21–0.40), moderate (0.41–0.60), substantial (0.61–0.80), and almost perfect (0.81–1.00)^15,18,21^. All the statistical analyses except power analysis were performed using the IBM SPSS (version 27; IBM Corp., Armonk, NY, USA). Statistical significance was set at *p* < 0.05.

## Results

### The differences between the overhead method, craniocaudal view, and isolated femoral anteroposterior view

As shown in Table 1, the difference between the overhead method and the isolated femoral anteroposterior view was significantly smaller than that between the craniocaudal view and the isolated femoral anteroposterior view in nail length (1.53 mm ± 1.26 vs.11.4 mm ± 3.45, *p* < 0.001) and in neck-shaft angle (5.82° ± 3.8 vs. 37.8° ± 5.7, *p* < 0.001). However, no significant differences existed in intertrochanteric length/femoral head diameter (0.23 ± 0.13 vs. 0.23 ± 0.13, *p* = 0.96) or the lateral condyle width/the medial condyle width (0.15 ± 0.16 vs.0.13 ± 0.08, *p* = 0.82).

**Table 1:** Difference between the overhead method and the isolated femoral anteroposterior view versus the difference between the craniocaudal view and the isolated femoral anteroposterior view.

Variables	Overhead-isolated		Craniocaudal-isolated		*p*-Value
Nail length, mm	1.53	(1.26)	11.4	(3.45)	< 0.001
Neck shaft angle, degree	5.82	(3.8)	37.8	(5.7)	< 0.001
Intertrochanteric length/femoral head diameter	0.23	(0.13)	0.23	(0.13)	0.96
lateral condyle length/medial condyle length	0.15	(0.16)	0.13	(0.08)	0.82

All data are presented as mean (SD).

### Intraclass correlation coefficient (ICC) and a fragment displacement to assess the overhead method

Table 2 shows the ICC results for the reliability of repeated measurements. ICC of the nail length, neck shaft angle, intertrochanteric length/femoral head diameter, and lateral condyle width/the medial condyle width was 0.96 (95% CI 0.847–0.996, *p* < 0.001, 0.96 (95% CI 0.822–0.995, *p* < 0.001), 0.98 (95% CI 0.932–0.998, *p* < 0.001), and was 0.99 (95% CI 0.938–0.998, *p* < 0.001), respectively. The maximum fragment displacement was 0.11 mm (2.4%) (Table 3).

**Table 2:** Intraclass correlation coefficient (ICC) for the reliability of repeated measurement.

Variables	ICC (95% CI)		*p*-Value
Nail length	0.96	(0.847–0.996)	< 0.001
Neck shaft angle	0.96	(0.822–0.995)	< 0.001
Intertrochanteric length/femoral head diameter	0.98	(0.932–0.998)	< 0.001
lateral condyle length/medial condyle length	0.99	(0.938–0.998)	< 0.001

**Table 3:** Free fragment displacement and free fragment width for each rat.

Rat no	Displacement (mm)	Free fragment width (mm)	Displacement/free fragment width (%)
1	0.06	4.39	1.4
2	0.04	4.43	0.9
3	0.05	4.17	1.2
4	0.01	4.01	0.3
5	0.11	4.67	2.4

## Discussion

In this experimental study, we developed a novel radiographic technique to repeatedly evaluate a live rat femur with a large segmental fragment. The novel aspect of this study was the development and comparison of the new overhead method technique with the traditional craniocaudal view. Our data indicated high intra-observer reliability and similarity to the isolated femoral anteroposterior view compared to the traditional craniocaudal view. The findings of this study could have important implications for future studies and clinical practices in this field.

The lateral view of the rat femur has already been established^22^. In animals, such as dogs, anteroposterior radiographs of the femur were established as the craniocaudal view^12^. However, this has not been established in rats. The craniocaudal view is challenging because a rat is small, and a rat’s knee and hip are strongly flexed and externally rotated in the living state. We could not extend and internally rotate them sufficiently with downward traction with the knees tightly together. The traditional craniocaudal view is also mentioned as less used for views of the stifle because of increased magnification^12^. However, we could lift the greater trochanter and make the femur more straightforward, lessening the angulation of the femur using this overhead method. Therefore, it is reasonable to use this overhead limb position.

Some studies using rat femurs have evaluated fracture healing and callus using bones isolated from sacrificed rats^10,23^. We believe we can reduce the number of rats needed when we continuously use this overhead method and lateral view to evaluate bone healing. This is crucial for laboratory animal ethics^24^. Furthermore, comparing data between rats is not feasible, as each is unique, even if they are kept under the same conditions. This method may provide a way to evaluate a single individual continuously and assess more accurately the bone-healing process, making it a valuable contribution to the field of orthopedics and fracture treatment.

The maximum fragment displacement between the first and third overhead imaging was 0.11 mm (2.4%). Although no method exists to determine the acceptable degree, we think it is acceptable in actual use. In addition to the fact that the degree of error of Osirix and the micro-computed tomography machine is unknown, we also have to consider our manual measurement errors. Considering these measurement errors, we expected this value to be microscopic.

Our study had certain limitations. First, the degree of leg traction varied slightly in the overhead method and craniocaudal view, necessitating adjustments for each case due to differences in leg stiffness and difficulty in extending the knee, particularly among rats of varying ages. However, we decided on the strength of the traction so that the rat’s buttocks floated 2 cm in the overhead method and showed its reliability. Second, the craniocaudal view was always obtained after three shots of the overhead method. Therefore, the rotation might have changed after surgery and at the time of the craniocaudal view. However, the displacement of the free fragment before the craniocaudal view remained within 0.11 mm, suggesting minimal or negligible rotation. Third, there is currently no established standard for determining an acceptable level of free fragment displacement that can be considered an excellent imaging method that does not stress the bone fragment. Therefore, the only way to determine this is by absolute values. However, this approach does not compensate for potential errors associated with manual measurement, Osirix, and micro-computed tomography machine errors. Fourth, all measurements in the study were conducted by a single examiner across three views, with a relatively small sample size. Nevertheless, the ICC values obtained were exceptionally high, exemplifying robustness in our findings. For instance, ICCs were 0.96 for both nail length and neck shaft angle, indicating that a sample size of 3 is adequate for precise estimations^25^. However, inter-observer reliability was not assessed with multiple examiners in this study. We acknowledge the necessity of measuring the inter-observer reliability of this method in future research endeavors. Fifth, our experiments were conducted solely on rats. The generalizability of our methods to similar animals, such as mice, remains to be established. Further research is essential to determine the applicability of our techniques in these contexts.

In conclusion, we developed the overhead method, a novel anteroposterior radiographic technique for the evaluation of a live rat femur. Our analysis suggested substantial intra-observer reliability and similarity to the isolated femoral bone compared to the traditional craniocaudal view. Although further studies are required to assess the inter-observer reliability of this method, the findings of this study could have important implications for future studies and clinical practices in this field.

## Data Availability

The data that support the findings of this study are available from the corresponding author upon reasonable request.
